# Was It a Case of Prolonged Gestation?

**Published:** 1883-08

**Authors:** S. F. Bennett

**Affiliations:** Richmond, Ill.


### Article IV.

### Was it a Case of Prolonged Gestation? By S. F. Bennett, m.d., Richmond, Ill.

On March 19th, I was called to Mrs. S., a fleshy, robust and plethoric woman, aged 24, and well advanced in her second pregnancy. She informed me that her last menstruation occurred in the latter part of the preceding June. She was positive as to the date, because certain circumstances had impressed it upon her memory that she was "all through" before the first day of July. The attack for which I was called was one of cerebral congestion. On account of the general plethora, and for certain other reasons, I advised a bleeding, but the patient had a dread of the lancet, and I temporized with potass. bromide, and informed her she might expect her labor about the 8th of April, as, following the old rule and counting back three months from June 30, and adding seven days, it would give us about the date I had indicated. The attacks of cerebral congestion becoming more frequent and severe, the patient submitted to venesection, April 29th, with the effect of relieving the head symptoms. The labor finally came on May 7th, and occupied eighteen hours, the first hint of its approach having been the sudden discharge of the waters, without the slightest antecedent pain. The presentation was normal, and she was delivered of a lusty ten-pound boy. Does not an analysis of this case show that it is probable that the term of Mrs. S.'s gestation must have exceeded 270 or even 280 days considerably?

---

### DURHÆMATOMA.

A woman, forty-two years of age, began to suffer from loss of memory and a depressed mental state. Later, the pupils enlarged, and she was slow in all her actions, but without motor disturbances. The vision became defective, especially the peripheric, and there was entire loss of smell. Death followed suddenly, with vomiting and collapse. The autopsy showed a hæmatoma in the dura mater, forming a sac, which was filled with liquid and coagulated blood on the right hemisphere, which latter was flattened and curved inward. The larger part of the central convolutions, and those of the frontal and temporal lobe, were compressed to a concave plane. It was remarkable that this compression in the motor region did not produce a more extended distribution of disease in the brain, but the coagulation of blood having only occurred after death, the pressure must have been not very great, the cerebro-spinal fluid being equally diffused, and flowing off partially in the venous sinus of the dura and partially through the lymphatic vessels of the head.—*Med. Chir. Centralblatt.*

